# Advancing Antiviral Design: Integrating Natural Products, Computation and Targeted Delivery

**DOI:** 10.1111/cbdd.70365

**Published:** 2026-07-23

**Authors:** Adedayo Ayodeji Lanrewaju, Abimbola Motunrayo Folami, Saheed Sabiu, Feroz Mahomed Swalaha

**Affiliations:** ^1^ Department of Biotechnology and Food Science, Faculty of Applied Science Durban University of Technology Durban South Africa

**Keywords:** antiviral drug discovery, computer‐aided drug design, machine learning, plant secondary metabolites, targeted drug delivery

## Abstract

The COVID‐19 pandemic highlighted the role of rapid viral mutation and global connectivity in accelerating viral emergence and spread, emphasising the necessity for expedited and adaptable antiviral drug discovery and development. Despite ongoing efforts to develop effective, low‐toxicity therapeutics, the number of antivirals that have achieved clinical approval remains limited. The shortfall is especially significant in developing countries, where access to new antivirals is limited by high prices, few options, import dependence, unstable supply chains and weak purchasing systems. The challenge is further heightened by the emergence of increasingly drug‐resistant variants while vaccines often provide inadequate protection against newly mutated or novel viruses. Consequently, the identification of novel antiviral agents that are both effective and cost‐efficient via innovative strategies for antiviral drug discovery is essential to manage and control viral infections. Therefore, this review examines the different challenges associated with conventional antiviral drugs alongside recent strategies in antiviral drug discovery and development, such as the exploitation of plant secondary metabolites with antiviral properties, advanced microscopy technologies, computer‐aided drug design, artificial intelligence and machine learning, gene‐editing technologies, drug combination therapy and nanotechnology‐enhanced drug delivery systems. Additionally, this study proposes a simple decision‐focused pathway integrating natural products, computation and targeted delivery to guide candidate prioritisation, optimisation and translation from discovery to implementation. While these emerging strategies offer considerable promise, challenges related to validation, toxicity, scalability and equitable access remain important considerations for successful clinical translation. Future research should therefore integrate complementary technologies to accelerate the development of effective antiviral agents against current and emerging viral threats.

## Graphical abstract

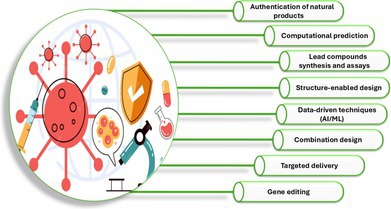

## 1 Introduction

Among the many instances of infectious disease outbreaks that humanity has faced, viral infections as exemplified by the COVID‐19 crisis undeniably provide the most significant current risk of a pandemic (Ali et al. 2021). Viruses exhibit complex molecular strategies that facilitate efficient replication and persistence within their host cells. Natural selection has provided them with the genetic ability to circumvent the host's defence mechanisms, ensuring the dissemination of their genetic materials and consequently their survival (Su et al. 2024). In addition, viruses present obstacles to broad‐spectrum antiviral medications that target both RNA and DNA viruses, which can be ascribed to factors such as their genomic structure (single or double genomic structure), site of replication (cytoplasm or nucleus) and the extent to which the virus relies on host proteins for its survival (Schoeman and Fielding 2019). Unfortunately, the occurrence of epidemic outbreaks caused by newly identified and recurring viruses presents a major public health risk (Lanrewaju et al. 2022), particularly in situations when vaccines and antiviral medications are unavailable.

Antiviral therapy continues to be the fundamental approach to treatment, with a wide variety of medicines such as amantadine, oseltamivir, nevirapine, tenofovir, acyclovir (Richman and Nathanson 2016) and vaccines such as twinrix, cervarix, fluzone, rotarix, imovax, varivax (Ellebedy and Ahmed 2016), which are accessible for some viral infections. Nevertheless, contemporary antiviral treatment encounters certain obstacles that hinder their efficient control of infections. The fact that more than 220 identified human viruses are in circulation, yet only few antiviral drugs have secured clinical approvals is a shortfall that raises significant concern (Cheung et al. 2024; Adamson et al. 2021), which becomes worrisome, especially in low‐income countries where expensive drugs are not easily accessible. To make matters worse, each virus needs specialised pharmaceutical interventions or therapies owing to their distinct characteristics and behaviours, necessitating customised treatments (Kausar et al. 2021). Examples include antiretroviral drugs (abacavir), neuraminidase inhibitors (influenza), hepatitis C (sofosbuvir, ledipasvir), herpes (acyclovir) among others. Unfortunately, the problem is worsened by drug‐resistant mutants, that greatly reduces the effectiveness of the antiviral agents, particularly when utilising inhibitors that target specific viral enzymes (Geretti et al. 2012; Locarnini and Yuen 2010). Therefore, the discovery of novel antiviral drugs that are both efficient and economical to manage viral infections effectively in cases when vaccines and conventional treatments are insufficient becomes imperative.

The advancement of therapeutically potent drugs for new viral diseases has required the use of very sophisticated molecules, yet the process of innovative antiviral drug development has been slow and hindered by several obstacles (Desselberger 2000), as it took over six decades for antiviral research to reach its present widespread status in contrast to antibiotics which achieved a targeted therapeutic level within three decades (Ali et al. 2021). Challenges include the identification of suitable viral and host targets, the limited availability of broad‐spectrum antivirals and the rapid emergence of viral mutations that can compromise therapeutic efficacy. In recent decades, intensive scientific investigations have led to the discovery of numerous synthetic antiviral medicines that demonstrate efficacy against various viral pathogens. Regrettably, these artificial substances have been shown to generate several detrimental consequences. These consequences may include neuropsychiatric complications, comprising a continuum of mild symptoms like irritability and sleep disruptions such as depression, psychosis, painful neuropathy and other side effects that may warrant terminating the therapy (Zareifopoulos et al. 2020). Occasionally, they may lose their effectiveness against newly developing virus strains that have developed resistance (Kurokawa et al. 2010). Furthermore, antiviral development is frequently constrained by lengthy development timelines, toxicity concerns, suboptimal pharmacokinetic properties and high attrition rates during preclinical and clinical evaluation. Given the global prevalence of viral infections, development of resistance to conventional drugs and the high expense of treatment, it is imperative to urgently devise novel approaches to identify inexpensive and efficient antiviral medications.

Antiviral techniques can be categorised as direct‐acting antivirals (DAAs), host‐targeted therapeutics (HTAs) and immune‐modulating approaches (Bekerman and Einav 2015; De Clercq and Li 2016). Direct‐acting antivirals obstruct viral proteins or replication mechanisms critical for the viral life cycle, while host‐targeted treatments disrupt cellular pathways utilised by viruses during infection (Bekerman and Einav 2015; Kaufmann et al. 2018). Conversely, immune‐modulating strategies augment or regulate host immune responses to promote virus control and illness outcomes (Iwasaki and Pillai 2014). Recent antiviral design strategies increasingly integrate these approaches, as illustrated by the development of dual PB2/JAK2 inhibitors that simultaneously suppress influenza viral replication and modulate host inflammatory responses (Yang et al. 2026). These complementary methodologies are the foundation of modern antiviral discovery and development, supporting other developing approaches examined in this study.

Previously, progress reports on antiviral drug discovery and development have been disseminated (Hangyu et al. 2024; Saxena et al. 2010, 2009), however less attention has been given to cutting edge techniques like computer‐aided drug design, artificial intelligence and machine learning. Moreover, existing reviews often discuss natural products, computational approaches and advanced delivery systems as separate strategies rather than components of an integrated antiviral development framework. Therefore, this review examines the different challenges associated with conventional antiviral drugs, alongside recent strategies in antiviral drug discovery and development, such as the exploitation of plant secondary metabolites with antiviral properties, advanced microscopy technologies, computer‐aided drug design, artificial intelligence and machine learning, gene‐editing technologies, drug combination therapy and nanotechnology‐enhanced drug delivery system. Obviously, a concise translational pathway linking natural product discovery, computational approaches and targeted delivery strategies is lacking. Consequently, this study outlines a simple, decision‐focused pathway integrating natural products, computation and delivery to guide choices from discovery through optimisation and translation prior another possible viral pandemic.

## 2 Limitations of Conventional Antiviral Agents

Conventional antiviral agents are constrained by limitations due to the rapid development of resistance, the persistence of latent infections, narrow spectrum activity, toxicity issues and high costs. Of these challenges, resistance due to rapid viral genetic change constitutes a significant challenge (Ghaebi et al. 2020). Viral genomes are characterised by rapid replication within host cells, which could result in the formation of an expanded gene pool in which new mutations emerge. The presence of antiviral drugs in these conditions exerts selection pressure, leading to the multiplication and spread of resistant viruses and consequently, the vulnerable population being replaced by the resistant viruses (Saxena et al. 2010). For instance, the K22 compound: (Z)‐N‐(3‐(4‐(4‐bromophenyl)‐4‐hydroxypiperidin‐1‐yl)‐3‐oxo‐1‐phenylprop‐1‐en‐2‐yl)‐benzamide showed strong anti‐coronavirus activity by blocking virus‐induced endoplasmic reticulum membrane zippering (Bills et al. 2023; Ricciardi et al. 2022), but was subsequently ineffective against certain human coronavirus 229E (HCoV‐229E) variants, known as escape mutants due to their resistance which is attributed to specific changes in the non‐structural protein 6 of the virus (Lundin et al. 2014). Beyond its use for SARS and MERS, lopinavir‐ritonavir was investigated for COVID‐19; however, human immunodeficiency virus (HIV) developed resistance to protease inhibitors including lopinavir‐ritonavir, early in its clinical use (Nastri et al. 2023; Zumla et al. 2016). Evasion from single‐target drugs has been demonstrated by resistance to nirmatrelvir in patients owing to increased viral mutation rates. In addition, the possibility of mutational signatures arising from treatment with molnupiravir has been highlighted and might be transmissible (Baniecki et al. 2025; Tamura et al. 2024; Sanderson et al. 2023). Furthermore, research has shown that although adamantanes and neuraminidase inhibitors (NAIs) are useful in managing influenza, nevertheless medication resistance resulting from I223V and S247 mutations renders them inappropriate for usage during the pandemic (Leung et al. 2024). Oseltamivir resistance via the neuraminidase H274Y mutation in H1N1 influenza A contributed to the dissemination of the pandemic H1N1 strain (Ait‐Aissa et al. 2018; Renzette et al. 2014). Notably, the therapy for chronic hepatitis B virus (HBV) infection has significantly progressed following the introduction of nucleoside analogue therapies (Lim 2017). Nevertheless, the extensive utilisation of less powerful nucleoside analogues with lower genetic thresholds, such as lamivudine and adefovir, has driven substantial resistance in the HBV, as demonstrated by rising cumulative resistance (European Association for the Study of the Liver 2012; Papatheodoridis et al. 2005).

Another significant challenge is viral latency, characterised by the persistence of viral genomes in host cells with minimal gene expression and no infectious particle production, which can subsequently reactivate to initiate productive replication (Speck and Ganem 2010). Most approved antivirals primarily exhibit virustatic properties and focus on active replication processes, resulting in limited efficacy against silent reservoirs (Saxena et al. 2010). Persistence of viruses can manifest as episomal DNA, as seen in herpesviruses or as an integrated provirus, characteristic of retroviruses, enabling the infection to remain clinically silent while maintaining the potential for resurgence when immune control diminishes or treatment is halted. Archived viral genomes may function as templates for renewed replication under favourable conditions, resulting in reactivation and clinical recurrence. De Leo et al. (2020) emphasised that viral episome maintenance proteins (EMPs) are crucial for maintaining episomal stability and facilitating long‐term persistence in viruses that retain their genomes as episomes. For example, HIV‐1 can establish a latent infection state resistant to antiretroviral therapy and host immune defences, with reactivation possible upon exposure of infected cells to activating stimuli (Siliciano and Greene 2011).

Moreover, the replication and transcription processes of positive‐stranded RNA viruses are intricate; hence, while designing drugs against viral infections, it is necessary to analyse their targets in detail. For instance, antiviral drug design often centres on critical core enzymes encoded by the virus, such as those linked to the replication‐transcription complex (RTC) and RNA‐dependent RNA polymerase (RdRp). This has resulted in the narrow‐spectrum activity of most antivirals designed to target unique viral proteins or life‐cycle stages leading to the susceptibility of humans to emerging viruses with no established therapeutics (Karim et al. 2023) and consequently the vulnerability of public health systems during outbreaks. Thus, to create novel and useful antiviral control techniques, a thorough grasp of the viral biochemistry enabled by characterising RTCs, namely their structure and activities is needed. In developed nations, initial ART selection for durable viral suppression is guided at diagnosis by HIV‐1 genotyping, which distinguishes acquired drug‐resistance mutations (DRMs) from transmitted resistant variants (tDRMs) (Bills et al. 2023; Saag et al. 2018). Thus, a detailed understanding of every potential target and the mechanism of action would provide researchers with information for the design and optimisation of broad‐spectrum antiviral therapeutics.

Another significant drawback in antiviral development is the cost, which disproportionately impacts patients in low‐income nations. For instance, Iyengar et al. (2016), evaluated hepatitis C drugs prices and affordability in 30 countries and reported a global variation and unaffordability. The variation is primarily influenced by patent and licensing restrictions that hinder generic competition, confidential pricing and discounts that diminish transparency, disparities in procurement and negotiation capabilities among countries and the lack of pooled purchasing mechanisms that could generate economies of scale. Unfortunately, developed nations like the United Kingdom are not left out of this problem; access to sofosbuvir for hepatitis C was delayed by about a year after its 2014 licensing because of pricing disputes (Henry 2018; Trooskin et al. 2015). Similarly, the long‐term viability of providing effective antiretroviral medication for HIV in Brazil is compromised by the unsustainably high cost associated with the advanced technology needed for its production and distribution, which could consequently impede patients' ability to get treated, thereby impacting the global endeavour to enhance the healthcare system (Benzaken et al. 2019).

Viruses are dependent on the host machinery for multiplication which might increase the risk of off‐target toxicity in host‐targeting approaches (Martin and Tripp 2024; Von Delft et al. 2023). The lack of selectivity in antiviral medications is a significant issue as it might lead to toxicity by interfering with the activity of normal cellular enzymes and DNA synthesis (Saxena et al. 2010). For human cytomegalovirus (HCMV), Minces et al. (2014), examined cidofovir and foscarnet in which different toxicities, including kidney injury, electrolyte imbalances and glycaemic effects were identified to be linked with foscarnet while kidney damage and neutropenia related to cidofovir (Biron 2006). Furthermore, favipiravir, authorised for treating influenza in Japan and investigated as a possible therapy for COVID‐19, was observed to increase uric acid in the bloodstream (Mishima et al. 2020).

In addition to resistance and toxicity, pharmacokinetic limitations continue to restrict the clinical effectiveness of many antiviral agents. Several approved antivirals exhibit suboptimal oral bioavailability, inadequate tissue penetration, rapid elimination or require parenteral administration, thereby reducing therapeutic efficiency and patient adherence. For example, acyclovir possesses relatively poor oral bioavailability, necessitating frequent dosing and prompting the development of the prodrug valacyclovir to improve systemic exposure (Wagstaff et al. 1994; De Clercq and Li 2016; Assis et al. 2021). Similarly, cidofovir requires intravenous administration and its clinical application is constrained by dose‐dependent nephrotoxicity (Biron 2006; Vora et al. 2017). Furthermore, inadequate drug penetration into viral sanctuary sites and tissue reservoirs may limit antiviral effectiveness and contribute to viral persistence despite treatment (Thompson et al. 2015; Fletcher et al. 2022). These pharmacokinetic challenges underscore the need for innovative drug delivery strategies capable of improving bioavailability, tissue‐specific targeting and sustained therapeutic concentrations. Collectively, these limitations highlight the need for next‐generation antiviral strategies that combine broad‐spectrum activity, reduced susceptibility to resistance, improved tissue penetration and bioavailability, lower toxicity and greater affordability to ensure effective and equitable management of both existing and emerging viral infections.

## 3 Recent Approaches in Antiviral Drug Discovery and Development

### 3.1 Exploitation of Plant Secondary Metabolites With Antiviral Properties

**FIGURE 1:**
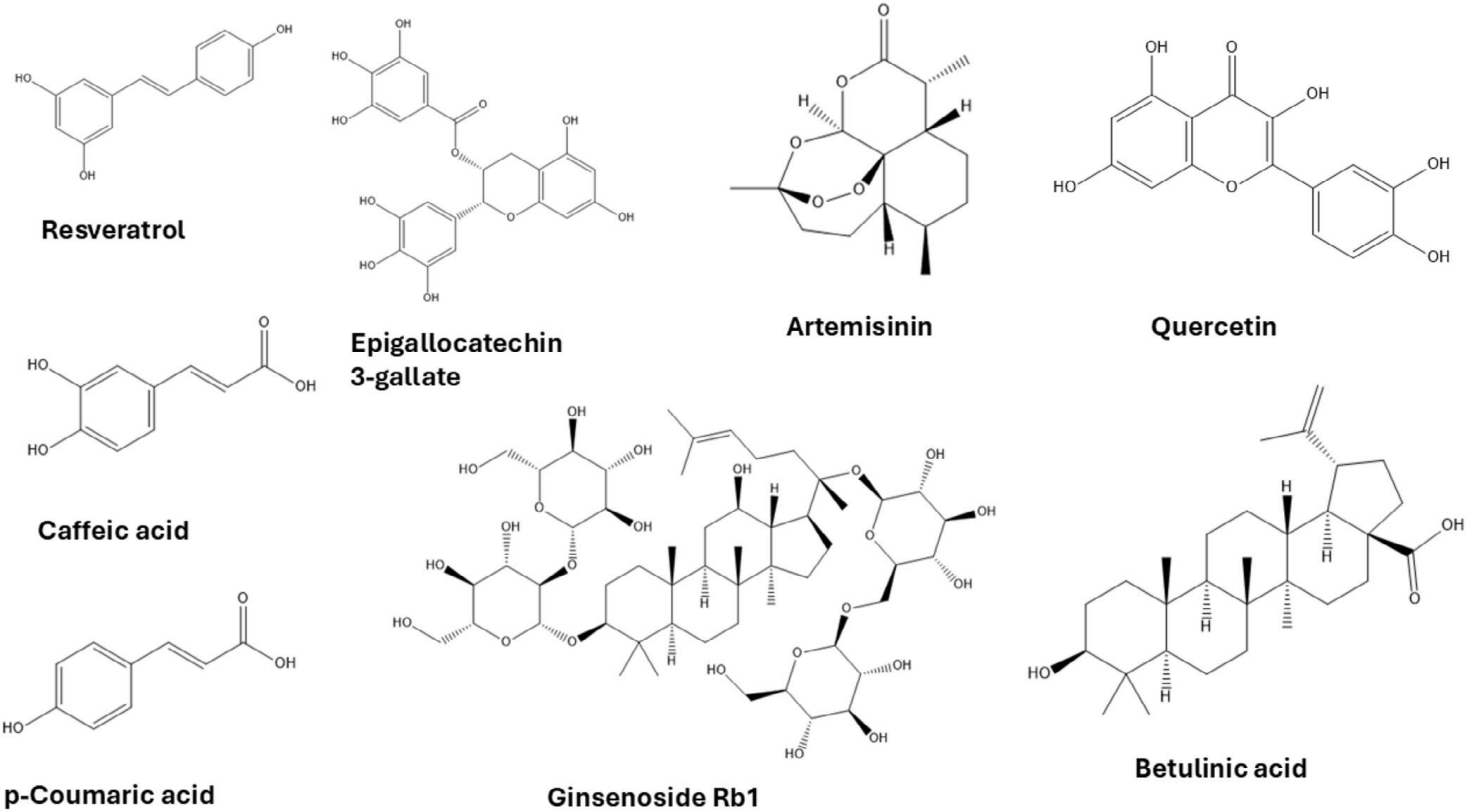
Chemical structures of some plant secondary metabolites with antiviral activities.

Plant secondary metabolites (PSMs) are structurally diverse small molecules generated through the secondary metabolic pathways (Yeshi et al. 2022; Pang et al. 2021). They have diverse roles in plant growth, development, innate immune defence (Piasecka et al. 2015), defence response signalling (Isah 2019) and adaptation to environmental challenges (Yang et al. 2018). Based on their biosynthetic roots, PSMs fall into major chemical groups including phenolics, terpenes, steroids, alkaloids and flavonoids. Presently, approximately 200,000 distinct PSMs have been catalogued and isolated (Kessler and Kalske 2018). Furthermore, PSMs possess significant roles, such as deterring pests and diseases, serving as communication signals that mediate plant‐microbe association and altering the microbial communities that are connected to hosts (Guerrieri et al. 2019). Additionally, they are accountable for the pigmentation, aroma and taste of plant‐based products. Throughout the years, they have been shown to have remarkable pharmacological potential and have been used as reservoirs of lead compounds with various biological activities for managing diverse diseases (Thirumurugan et al. 2018). They could be used to induce therapeutic effects in humans by functioning as neurotransmitters, hormones, endogenous metabolites, signalling molecules and ligands among others (Bilal et al. 2017). Moreover, the antiviral properties documented for some PSMs support their evaluation as alternatives for preventing and managing viral infections owing to their reduced or minimal toxicity relative to conventional antiviral drugs. Figure 1 shows the chemical structures of some PSMs with antiviral activities. These metabolites have been adopted for different antiviral effects which include: (1) preventing virus entry and attachment (Derksen et al. 2016; Helfer et al. 2014), (2) inhibiting viral replication (Ebenezer et al. 2019), (3) inhibiting protein synthesis (Oloche et al. 2023; Mansouri et al. 2012), (4) modulating the host's immune system (Alhazmi et al. 2021), (5) modulating cellular signalling pathways (Bhuiyan et al. 2020) and (6) directly killing the viruses (Bhuiyan et al. 2020; Perez 2003). These antiviral activities are mediated through interactions with both viral targets, including polymerases, proteases, integrases and envelope proteins, as well as host signalling pathways involved in viral entry, replication and immune responses.

Sodium ferulate, the sodium salt of ferulic acid found in medicinal plants such as *Angelica sinensis* and *Ligusticum chuanxiong*, was reported to inhibit influenza virus replication by engaging toll‐like receptors TLR7 and TLR9, which in turn drive interferon regulatory factor 7 (IRF7) into the nucleus, enhancing type‐1 interferons production (Zhu et al. 2019). This mechanism suggests that sodium ferulate exerts antiviral activity primarily through host‐directed immune modulation rather than direct viral inhibition. Also, chicoric acid, a phenolic compound abundant in *Cichorium intybus* (chicory) and *Echinacea purpurea* , inhibits HIV‐1 integrase, the enzyme that catalyses insertion of viral DNA into the host genome (Robinson et al. 2000). By targeting this essential step, chicoric acid interferes with proviral establishment and subsequent viral propagation. Further mechanistic evaluation showed that l‐chicoric acid inhibited HIV‐1 integration at concentrations ranging from 500 nM to 10 μM, although concentrations above 1 μM were also associated with inhibition of viral entry, indicating that its anti‐HIV activity may involve more than integrase inhibition alone (Reinke et al. 2004). Conversely, gallic acid exhibits anti‐HSV‐2 activity by preventing virion attachment to host cell receptors, thereby limiting its spread (Kratz et al. 2008). Furthermore, the immunomodulatory activities of glycyrrhizin via interferon‐gamma induction and its anti‐inflammatory mechanisms have been reported (Richard 2021; Fiore et al. 2008). Also, it inhibits severe acute respiratory syndrome‐associated coronavirus (SARS‐CoV) replication and prevents the adsorption and penetration of the virus. In SARS‐CoV‐infected Vero cells, glycyrrhizin showed an EC_50_ of 300 mg/L, a CC_50_ > 20,000 mg/L and a selectivity index > 67, supporting measurable antiviral activity but also indicating that relatively high concentrations were required for inhibition (Cinatl et al. 2003). These findings indicate that glycyrrhizin can interfere with both viral entry and post‐entry replication events. Interestingly, at a minimal concentration of 0.06%, isoborneol was reported to completely halt herpes simplex virus (HSV‐1) replication without marked cytotoxicity within the tested concentration range of 0.016%–0.08% (Armaka et al. 1999). In addition, β‐pinene and limonene showed potent anti‐HSV‐1 effects, producing a 100% loss of viral infectivity via direct contact with free virions at 10 and 25 μg/mL, respectively (Astani and Schnitzler 2014). Remarkably, celastrol reportedly inhibits the replication of dengue virus (Yu et al. 2017), human immunodeficiency virus (Narayan et al. 2011) and hepatitis C virus (Tseng et al. 2017), highlighting its potential as a broad‐spectrum antiviral scaffold acting against multiple viral families. For dengue virus, celastrol inhibited DENV‐1, DENV‐2, DENV‐3 and DENV‐4 RNA replication with EC_50_ values of 0.19 ± 0.09, 0.12 ± 0.11, 0.16 ± 0.14 and 0.17 ± 0.08 μM, respectively (Yu et al. 2017). Similarly, against HCV, celastrol inhibited viral replication in HCV subgenomic and HCVcc infection systems with EC_50_ values of 0.37 ± 0.022 and 0.43 ± 0.019 μM, respectively (Tseng et al. 2017). Table 1 gives a summary of different PSMs and the corresponding mode of action through which their antiviral activity was exerted against viruses. To facilitate interpretation of the available evidence, this table categorises the cited studies according to their level of experimental validation, including in silico prediction, biochemical assays, cell‐based antiviral assays and in vivo validation where available. This distinction is important because the reported antiviral activities of PSMs are supported by varying levels of evidence. Computational approaches, including molecular docking and molecular dynamics simulations, play an important role in antiviral discovery by enabling the rapid screening of large numbers of PSMs, identifying potential molecular targets and prioritising promising candidates for further investigation. However, because these approaches represent an early stage of the drug discovery process, additional biochemical, cellular and in vivo studies are generally required to confirm antiviral efficacy, safety and translational potential.

**TABLE 1:** Plant secondary metabolites with antiviral activity.

Plant secondary metabolites	Virus	Mode of action	Level of experimental validation	References
Caffeic acid (CA)	Influenza A virus (IAV)	Viral multiplication is suppressed in vitro by CA binding to proteins associated with replication	Cell‐based antiviral assay	Utsunomiya et al. (2014)
	Herpes simplex virus type 1 (HSV‐1)	During the early phase of viral DNA replication, CA inhibits viral multiplication in vitro by binding to key replication proteins	Cell‐based antiviral assay	Zhou et al. (2023), Ikeda et al. (2011)
	Ilheus virus	From a molecular docking study, CA interacts with the allosteric site on the Ilheus virus envelope protein, potentially disrupting its function	In silico prediction (molecular docking)	Saivish et al. (2023)
Oleanolic acid (OA)	Hepatitis C virus (HCV)	As a noncompetitive inhibitor, OA partially inhibits HCV NS5B RNA‐dependent RNA polymerase to hinder replication	Biochemical assay	Kong et al. (2013)
	IAV	By disrupting hemagglutinin and sialic acid receptor interactions, OA blocks virus and host cell contact	Cell‐based antiviral assay	Shao et al. (2023)
	Dengue virus (DENV)	Molecular docking analysis reveals the marked affinity of OA for the non‐structural proteins NS1 and NS5 of DENV	In silico prediction (molecular docking)	Kaushik et al. (2021)
	HSV‐1	By destabilising UL8, a part of the helicase‐primase complex, OA disrupts the machinery essential for genome replication	Cell‐based antiviral assay	Shan et al. (2021)
Betulinic acid (BA)	DENV	Following entry, BA limits dengue replication by suppressing viral RNA and protein synthesis	Cell‐based antiviral assay	Loe et al. (2020)
Dammarenolic acid	Human immunodeficiency virus type 1 (HIV‐1)	Dammarenolic acid inhibits HIV‐1 replication and viral infectivity	Cell‐based antiviral assay	Esimone et al. (2010)
Quercetin	HCV	Quercetin inhibits the virus replication by hindering oxidative/nitrosative stress and modifying PI3K‐LXRα‐driven lipogenesis associated with steatosis development and progression of the virus	Cell‐based antiviral assay	Septembre‐Malaterre et al. (2022), Pisonero‐Vaquero et al. (2014)
	DENV	Quercetin inhibits viral RNA polymerase activity, hence obstructing the replication of DENV‐2	Cell‐based antiviral assay	Singh et al. (2023)
	Rotavirus‐SA11 (RV‐SA11)	Quercetin inhibits viral replication and lowered expression of viral proteins and titre via the induced early activation of NF‐κB pathway of RV‐SA11	Cell‐based antiviral assay	Banerjee et al. (2022)
	Severe acute respiratory syndrome coronavirus 2 (SARS‐CoV‐2)	Quercetin 3‐O‐rhamnoside exhibited the lowest binding free energy by binding to the active site residues (His41, Cys145, Glu166 and Gln189) of SARS‐CoV‐2 Mpro in a molecular dynamics (MD) simulation experiment	In silico prediction (molecular docking and MD simulation)	Lanrewaju et al. (2024)
Artemisinin	SARS‐CoV‐2	Artemisinin binds to the Mpro of the virus via one of the catalytic residues, Cys145, thereby hindering the activity of the protein in silico	In silico prediction (MD simulation)	Badraoui et al. (2022)
	IAV	Artemisinin and derivatives inhibit the activity of PDE4, leading to an accumulation of intracellular cAMP and subsequently reducing the ERK/MAPK signalling and preventing the nuclear export of IAV viral ribonucleoprotein (vRNP)	Cell‐based antiviral assay	Yang et al. (2023)
Taxifolin (TA)	HCV	By targeting the hepatocyte CD81 receptor, an essential gateway for viral uptake into cells, TA has the potential to prevent HCV entry as detailed in a molecular docking analysis	In silico prediction (molecular docking)	Dey et al. (2023), Liu et al. (2023)
	HIV	During the earliest attachment/entry phase, TA suppresses HIV‐1 to suppress initial infection events	Cell‐based antiviral assay	Yang et al. (2021), Zheng et al. (2021)
	SARS‐CoV‐2	As an inhibitor of the Mpro of SARS‐CoV‐2, TA shows promising potential	In silico prediction (molecular docking and MD simulation)	Fischer et al. (2020)
	Ebola virus	In docking and simulation analysis, TA binds strongly to the Ebola virus VP35 protein, establishing numerous hydrogen bonds	In silico prediction (molecular docking and MD simulation)	Badshah et al. (2021)
Kaempferol	Hepatitis B virus (HBV)	From a molecular docking analysis, kaempferol interfered with HBV by disabling the activity of HBV polymerase and core proteins	In silico prediction (molecular docking)	Parvez et al. (2021)
	Varicella‐zoster virus (VZV)	Kaempferol inhibits the viral life cycle at the DNA replication stage, thereby stopping the infection	Cell‐based antiviral assay	Park et al. (2022)
	SARS‐CoV‐2	In SARS‐CoV‐2, kaempferol binds the heptad‐repeat segment of the S2 subunit to inhibit membrane fusion and engages protein 5R84 via hydrogen bonds with key residues	In silico prediction (molecular docking), Cell‐based antiviral assay, In vivo validation	Gao et al. (2023), Sun et al. (2023)
Naringenin (NGN)	SARS‐CoV‐2	Against SARS‐CoV‐2, NGN has the potential to interfere with viral replication by targeting key non‐structural proteins, including NSP12 (RNA‐dependent RNA polymerase), NSP7, NSP8 and NSP3, thereby disrupting the viral replication and transcription machinery	In silico prediction (molecular docking and MD simulation)	Aleebrahim‐Dehkordi et al. (2023)
	Hepatitis C virus (HCV)	For HCV, NGN hinders proliferation by obstructing the formation of infectious virions before release	Cell‐based antiviral assay	Goldwasser et al. (2011)
	Zika virus	In Zika infection, NGN may act late in the viral cycle, as a non‐competitive blocker of the NS2B‐NS3 protease	Cell‐based antiviral assay	Cataneo et al. (2019)
Rosmarinic acid (RA)	Japanese encephalitis virus (JEV)	In the brain, RA inhibits the multiplication of JEV and dampen secondary inflammation responses driven by microglial activation	In vivo validation	Swarup et al. (2007)
	Enterovirus A71	Against EnterovirusA71, RA disrupts virion engagement by interfering with virus‐P‐selectin glycoprotein ligand‐1 (PSGL1) and heparan sulphate chains on the cell surface	Cell‐based antiviral assay	Hsieh et al. (2020)
	IAV	For IAV, RA blocks entry and replication by lowering GSK3β and phosphorylated AKT levels	Cell‐based antiviral assay	Jheng et al. (2022)
	DENV	In DENV, RA binds strongly to the envelope domain III (EDIII) protein, present in all four serotypes of the virus	Cell‐based antiviral assay	Panchal et al. (2022)
*p*‐Coumaric acid	H1N1 influenza virus	H1N1 influenza virus was prevented from attaching to the host cells during the adsorption stage by hindering viral surface proteins from engaging their host‐cell receptors thereby stopping attachment and accompanying infection	Cell‐based antiviral assay	Kim and Chung (2018), Chojnacka et al. (2021)
Baicalein	JEV	Inhibit JEV multiplication by accumulating within host cells. Additionally, it has the potential to hinder the attachment and entrance of viruses, attach to viral RNA or engage with key JEV proteins	Cell‐based antiviral assay	Johari et al. (2012)
	DENV	It impedes intracellular replication of the virus, deactivating unbound DENV particles by blocking their binding to host membranes	Cell‐based antiviral assay	Moghaddam et al. (2014)
	SARS‐CoV‐2	This metabolite suppresses the activity of main protease (Mpro) and 3‐chymotrypsin‐like proteases (3CLpro)	In silico prediction (molecular docking), cell‐based antiviral assay, in vivo validation	Liu et al. (2021), Song et al. (2021)
Excoecarianin	HSV‐2, HCV	Impedes the first stage of HSV‐2 replication and diminishes the virus's ability to infect, hence preventing HSV‐2 infection	Cell‐based antiviral assay	Cheng et al. (2011)
Resveratrol	SARS‐CoV‐2, Middle East respiratory syndrome coronavirus (MERS‐CoV)	Resveratrol inhibits viral replication by suppressing post‐entry replication processes, reducing viral protein expression and attenuating virus‐induced cellular damage and apoptosis	Cell‐based antiviral assay	Yang et al. (2020), Lin et al. (2017)
Spiraeoside	SARS‐CoV‐2, Rotavirus A	Spiraeoside exhibited potential antiviral activity by binding to the RNA dependent RNA polymerase of both viruses in molecular docking and MD simulation analysis	In silico prediction (molecular docking and MD simulation)	Lanrewaju et al. (2024a)
Apigenin‐4′‐glucoside, gnetin L, (2S)‐6‐(gamma,gamma‐dimethylallyl)‐3′,4′‐dimethoxy‐6″,6″‐dimethylpyran[2″,3″:7,8]flavanone	Rotavirus A	The trio showed potential broad spectrum antiviral action against RVA by binding to the viral proteins 7 (VP7A, VP7C and VP7D) of the virus in silico	In silico prediction (molecular docking and MD simulation)	Lanrewaju et al. (2024b)

Despite their remarkable potential antiviral activities, their advancement as medicines may be limited by low solubility and bioavailability and in some cases toxicity, which results after bioactivation (Giordano et al. 2023; Ponticelli et al. 2023). In addition, considerable variability exists in the reported antiviral potency of phytochemicals, and many candidates have not progressed beyond preliminary computational, biochemical or in vitro evaluation, thereby limiting assessment of their translational potential. The biotransformation of PSMs has led to debate over the years with high molecules along with tannins, flavonoids and their glycosides have the potential to be adsorbed and bio‐transformed by intestinal microbiota. Interestingly, various derivatisations have demonstrated effectiveness in enhancing their solubility and minimising toxicity (Owen et al. 2022). Additionally, metabolic activation of various terpenoids can yield toxic intermediates making it essential to assess the safety of PSMs and their biotransformation products (Wen and Gorycki 2019). Moreover, polyphenols have been reported to interact with multiple protein targets and may exhibit broad‐spectrum properties, which can complicate accurate characterisation of their mechanisms of action and is often attributed to nonspecific, low‐selectivity binding. This phenomenon is often driven by assay artefacts such as photoactivated reactivity, redox cycling, metal chelation, assay technology interferences, membrane disruption and other factors contingent upon the presence of many substructures within the interfering molecules classified as PAINS (pan‐assay interference compounds). Consequently, several guidelines have been outlined to assist researchers in minimising both financial and time loss towards investigating their bioactivity (Bisson et al. 2016; Baell and Walters 2014). Consequently, PAINS‐related concerns should be carefully considered during study design, and antiviral activities attributed to these compounds should be confirmed using multiple orthogonal assays before advancing to more resource‐intensive investigations. Conversely, some reports opined that some molecules categorised as PAINS do not necessarily exhibit high assay promiscuity (Capuzzi et al. 2017; Dimova et al. 2017). Thus, PAINS classification should be interpreted cautiously and evaluated on a case‐by‐case basis rather than applied indiscriminately (Aldrich et al. 2017). Therefore, future investigations must prioritise enhancing the solubility and bioavailability of PSMs via derivatisation and delivery systems. In addition, their biotransformation and safety profiles should be examined via metabolomics and microbiome investigations while validating their antiviral activity via rigorous orthogonal assays.

### 3.2 Advanced Microscopy Technologies

Microscopy is an essential tool in contemporary bio‐medical research as the only technique capable of accurately analysing the intricate spatial and temporal changes in living systems by providing the highest resolution necessary to depict biological systems in the most realistic manner (Cortese and Laketa 2021). The primary benefit of high‐throughput microscopy in drug discovery, as opposed to traditional biochemical methods, is its ability to utilise cellular assays that are more accurate in predicting in vivo conditions, especially compound uptake and intracellular metabolism. Furthermore, the capacity to multiplex several read‐outs in a single test enables the evaluation of toxicity, mechanisms of action and multiple drug profiling, thereby eliminating reliance on secondary and tertiary testing (Simm et al. 2018). Electron microscopy has long been the primary method for direct visualisation of viruses. Although, super‐resolution microscopy now enables nanoscale mapping of viral‐protein distribution on the virion surface (Muranyi et al. 2013; Chojnacki et al. 2012), EM remains unrivalled for detailed structural and morphological insights and was pivotal in viral research, particularly in 2020 to investigate SARS‐CoV‐2 infection and pathogenesis during the pandemic (Cortese and Laketa 2021). Specifically, cryo‐EM offers near‐atomic resolution that links macromolecular architecture to function in viral complexes. For instance, the first cryo‐EM structure of the trimeric spike ectodomain of SARS‐CoV‐2, with a resolution of 3.5 Å was deposited in the Protein Data Bank (PDB) in late February 2020 under two months following the publication of the first genome sequence of SARS‐CoV‐2 (Wrapp et al. 2020).

Precise high‐resolution structures perform critical responsibilities in directing, facilitating rational drug design and elucidating the functional roles of bioactive molecules. The structure of the SARS‐CoV‐2 replication and transcription complex (RTC) consisting of the viral RNA‐dependent RNA polymerase (RdRp) nsp12 and several non‐structural proteins was determined using Cryo‐EM by analysing the compound formed between an RNA template and the nucleoside analogue remdesivir; an antiviral drug that can block viral replication (Yin, Mao, et al. 2020). Therefore, this technique has facilitated drug design guided by the viral structure and produced therapeutic antibodies that bind in the femtomolar range and neutralise at picomolar levels (Schoof et al. 2020).

In addition to structural characterisation, advanced microscopy technologies have become integral tools throughout the antiviral drug discovery and development pipeline. High‐content imaging (HCI) platforms combine automated microscopy with quantitative image analysis to enable the simultaneous assessment of viral replication, host‐cell responses, cytotoxicity and compound efficacy in virus‐infected cells (Simm et al. 2018). Unlike conventional target‐based biochemical assays, image‐based phenotypic screening provides a more physiologically relevant evaluation of antiviral activity by enabling the simultaneous assessment of viral replication, host‐cell responses and compound‐induced phenotypic changes within infected cells (Pegoraro and Misteli 2017).

The practical value of advanced microscopy in antiviral discovery is demonstrated in some studies. For instance, cryo‐electron microscopy was instrumental in resolving the prefusion structure of the SARS‐CoV‐2 spike glycoprotein shortly after the emergence of COVID‐19, providing a structural framework that accelerated the development of vaccines, neutralising antibodies and viral entry inhibitors (Wrapp et al. 2020). Similarly, cryo‐EM analysis of the SARS‐CoV‐2 RNA‐dependent RNA polymerase in complex with remdesivir revealed the structural basis of antiviral inhibition, thereby facilitating structure‐guided optimisation of antiviral therapeutics (Yin, Mao, et al. 2020). In addition, super‐resolution fluorescence microscopy has been employed to investigate the nanoscale organisation of HIV‐1 envelope proteins during virion maturation, providing mechanistic insights into viral infectivity and potential antiviral intervention strategies (Chojnacki et al. 2012). These examples demonstrate how advanced microscopy technologies can directly contribute to antiviral target identification, mechanism elucidation and rational drug design.

Advanced microscopy also contributes to elucidating antiviral mechanisms of action. Live‐cell fluorescence microscopy and super‐resolution imaging allow the real‐time visualisation of viral attachment, entry, intracellular trafficking, genome replication, assembly and egress, thereby enabling researchers to determine the specific stages of the viral life cycle targeted by candidate antiviral compounds (Chojnacki et al. 2012; Muranyi et al. 2013). Furthermore, electron microscopy has been used to evaluate virus‐induced membrane rearrangements and replication organelles, providing direct evidence of antiviral effects on viral replication complexes and host‐cell ultrastructure (Cortese et al. 2020; Wolff et al. 2020). Beyond compound screening and mechanistic studies, microscopy technologies are increasingly employed to evaluate advanced antiviral drug‐delivery systems. Confocal microscopy, fluorescence microscopy and electron microscopy can be used to track the cellular uptake, intracellular localisation and intracellular release of nanoparticle‐based antiviral formulations, thereby supporting the optimisation of targeted drug delivery and improving therapeutic efficacy while minimising off‐target effects. For example, confocal fluorescence microscopy was used to monitor the intracellular uptake and sustained release of antiretroviral‐loaded poly(lactide‐co‐glycolic acid) nanoparticles in HeLa cells, demonstrating their potential as long‐acting antiviral delivery systems (Mandal et al. 2015).

Research on the cellular ultrastructural alterations triggered by SARS‐CoV‐2 has been crucial in comprehending the virus's effects on infected cells and has offered insights into possible treatment approaches (Cortese et al. 2020; Ogando et al. 2020; Wolff et al. 2020). Remarkably, the advanced microscopy techniques that have been developed are not specifically designed for SARS‐CoV‐2; instead, they can illuminate the viral pathophysiology with a distinct advantage for being able to act as a first line investigative tool when novel pathogens emerge. However, the main obstacle preventing the widespread use of microscopy technologies is a need for highly skilled technical experts to accurately identify the ultrastructural morphologies of viruses, while the resulting images need to be interpreted carefully as well, to avoid ambiguity. In addition, the acquisition, maintenance and operation of advanced imaging platforms such as cryo‐electron microscopy and super‐resolution microscopy require substantial financial investment and specialised infrastructure, which may limit their accessibility, particularly in resource‐constrained settings.

### 3.3 Computer‐Aided Drug Design (CADD)

Currently, there is a growing interest in CADD methods owing to their capacity to mitigate constraints of throughput, timelines and expenditure associated with conventional experimental techniques. CADD encompasses the adoption of computational techniques to identify potential druggable targets, virtually screen massive chemical libraries for potent hits, optimise lead compounds and analyse their potential toxicity. Therefore, it narrows the pool of compounds requiring laboratory validation while raising the odds of success by excluding candidates likely to be inactive or harmful at an early stage (Segall and Barber 2014). So far, CADD has already contributed to the delivery of approved new therapeutics including HIV‐1 protease inhibitors atazanavir (Robinson et al. 2000), saquinavir (Krohn et al. 1991), indinavir (Chen et al. 1994) and ritonavir (Kempf et al. 1995), anti‐cancer agent raltitrexed (Anderson 2003) and the antibiotic norfloxacin (Rutenber and Stroud 1996).

In order to make CADD more efficient and accurate, new methods have been designed and combined (Vamathevan et al. 2019) as structure‐based drug discovery (SBDD) (Leach and Hann 2007) and ligand‐based drug discovery (LBDD) (Vidal et al. 2011), which are the two distinct methodologies. Selecting an appropriate CADD strategy is largely governed by whether high‐resolution structural data exist for the target protein. Principal approaches utilised in CADD encompass virtual screening (structure‐ and ligand‐based), molecular docking and molecular dynamics (MD) simulations. Molecular docking predicts the preferred orientation and binding interactions of a ligand within the binding domain of a target protein, offering a preliminary assessment of binding affinity and aiding in the selection of potential compounds for subsequent exploration (Pagadala et al. 2017). Virtual screening, whether structure‐based or ligand‐based, facilitates the swift assessment of thousands to millions of compounds from chemical libraries to pinpoint molecules with favourable pharmacological properties and target specificity (Lionta et al. 2014). Subsequent to docking and MD simulations, are commonly utilised to evaluate the stability, conformational behaviour and dynamic interactions of protein‐ligand complexes under physiologically relevant settings. In contrast to docking, which offers a static depiction of binding, MD simulations incorporate molecular flexibility and temporal variations, hence enhancing the accuracy of binding predictions and hit prioritisation (Hollingsworth and Dror 2018). Collectively, these synergistic methods form the basis of contemporary CADD workflows and have significantly expedited antiviral drug discovery. For SBDD, an experimentally determined structure from nuclear magnetic resonance or X‐ray is required (Leach and Hann 2007). When an experimental structure is unavailable, the three‐dimensional fold of the target can be inferred via homology modelling or ab initio prediction (Cavasotto and Phatak 2009; Wu et al. 2007). Thereafter, molecular docking and structure‐guided virtual screening are conducted once the structure is obtained (Kitchen et al. 2004). When a dependable three‐dimensional structure cannot be produced, either through computational modelling or experimental determination, the LBDD approach is frequently utilised as a substitute.

Using an optimised homology model of the chikungunya virus (CHIKV) NSP2 protease alongside a pharmacophore model. Bassetto et al. (2013), virtually screened a library of commercially available molecules which resulted in several promising drug candidates that inhibited CHIKV‐induced cytopathic effect formation in vero cells effectively, even at low micromolar concentrations. Furthermore, a recent in silico investigation docked metabolites from corn silk and *Crescentia cujete* against spike glycoproteins of wild‐type and omicron SARS‐CoV‐2 strains (Aribisala et al. 2022). The five best scoring ligands were subsequently carried forward into 100 ns molecular dynamics (MD) simulation, which confirmed that several PSMs display broader spectrum activity and stronger binding than the reference drug zafirlukast. Also, identified lead compounds formed stable complexes with the investigated targets except kaempferol‐7‐glucoside. Table 2 shows a summary of commercialised antiviral drugs into which some form of computational technique was integrated into their discovery process.

**TABLE 2:** Antiviral drugs that adopted computer‐aided drug design (CADD) in the discovery process.

Drug	Target	CAAD contribution to the discovery	References
Saquinavir	Human immunodeficiency virus (HIV) protease	SBDD (TSMC)	Leelananda and Lindert (2016), Van Drie (2007)
Indinavir	HIV protease	SBDD (TSMC driven by molecular modelling and X‐ray crystal structure)	Van Drie (2007)
Ritonavir	HIV protease	SBDD, LBDD, SAR and lead optimisation	Lyle (2007), Van Drie (2007), Kempf et al. (1998)
Delavirdine	HIV reverse transcriptase	SBDD (Virtual screening against HIV‐1 RT) and LBDD (Lead optimisation and SAR)	Adams et al. (1998)
Nelfinavir	HIV‐1 protease	LBDD (Iterative protein cocrystal structure analysis and lead optimisation), Iterative SBDD and TSMC	Lyle (2007), Wlodawer and Vondrasek (1998), Kaldor et al. (1997)
Efavirenz	Non‐nucleoside HIV reverse transcriptase inhibitor	SBDD (Screening compounds against HIV‐1 RT through computational dissimilarity analysis and lead structure optimisation)	Lyle (2007)
Amprenavir	Antiretroviral (HIV‐1) protease	SBDD, LBDD, protein modelling and molecular dynamics	Batool et al. (2019), Leelananda and Lindert (2016), Lyle (2007)
Oseltamivir	Influenza A and B neuraminidase	Rational drug design via high‐resolution X‐ray crystal structures and the transition state analogue SBDD	Lew et al. (2000)
Zanamivir	Influenza neuraminidase	SBDD (Active site modelling)	Talele et al. (2010), Clark (2006), Von Itzstein et al. (1993)
Lopinavir	HIV‐1 protease	SBDD (TSMC) [3D modelling and docking]	Sham et al. (1998)
Atazanavir	HIV‐1 protease	Computational mapping of protein binding sites and ligands docking	Athanasiou and Cournia (2018)
Enfuvirtide	HIV‐1 protease	Homology modelling	Athanasiou and Cournia (2018)
Fosamprenavir	HIV‐1 protease	Structure‐based design	Lyle (2007)
Tipranavir	Nonpeptidic HIV‐1 protease	Monte Carlo based automatic docking, X‐ray crystallography, lead optimisation	Lyle (2007)
Darunavir	Nonpeptidic HIV‐1 protease	SBDD and LBDD	Ghosh et al. (2007)
Raltegravir	HIV‐1 integrase	MD and flexible ligand docking	Schames et al. (2004)
Boceprevir	Hepatitis C virus (HCV)	SBDD (TSMC and SAR) using X‐ray crystal structures and SAR optimisation	Njoroge et al. (2008)
Rilpivirine	HIV Non‐nucleoside reverse transcriptase	SBDD (homology modelling, compound screening, docking, mechanistic studies and transition state isostere modelling)	Lyle (2007)
Telaprevir	HCV NS3/4 A protease	Substrate‐based inhibitor design and structure‐based inhibitor optimisation	Rao et al. (2015)
Dolutegravir	HIV‐1 Integrase	PBDD (two‐metal binding pharmacophore structural‐based design)	Bailly and Cotelle (2015), Kawasuji et al. (2013)
Grazoprevir	HCV NS3/4 A protease	Molecular modelling and docking	Athanasiou and Cournia (2018)

Abbreviations: HTS, high throughput screening; LBDD, ligand‐based drug design; MD, molecular dynamics; PBDD, pharmacophore‐based drug design; SAR, structural activity relationship; SBDD, structure‐based drug design; TSMC, transition‐state mimetic concept.

Generally, the influence of computational drug discovery is significant with increasing utilisation and advancements in virtual screening and docking workflows, which still offers rough approximations. To further refine the prediction of protein‐ligand interactions, MD simulations are often integrated with free‐energy calculation methods such as molecular mechanics Poisson‐Boltzmann surface area (MM‐PBSA) and molecular mechanics generalised Born surface area (MM‐GBSA), which provide more reliable estimates of binding affinity and complex stability (Genheden and Ryde 2015). Nonetheless, these methodologies depend on classical force fields and may not entirely encompass quantum‐level electronic phenomena. To overcome this constraint, quantum mechanical approaches, especially density functional theory (DFT), have been progressively integrated into drug development processes to elucidate electronic interactions, reaction mechanisms and intermolecular forces (Ginex et al. 2024). Such methodologies can yield insights that enhance experimental research and improve confidence in computational predictions.

### 3.4 Artificial Intelligence (AI) and Machine Learning (ML)

**FIGURE 2:**
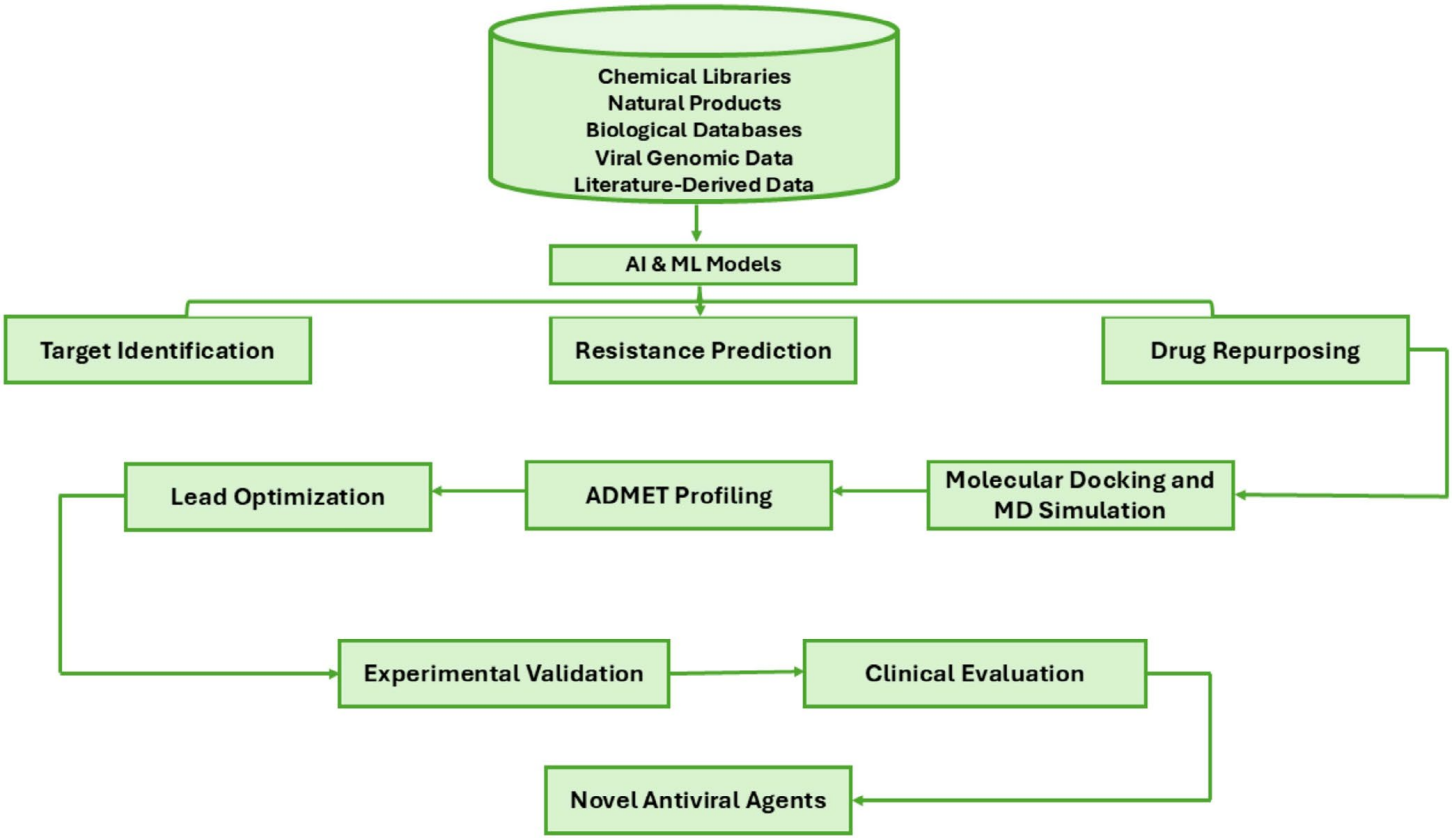
Applications of artificial intelligence and machine learning in antiviral drug discovery and development. Diverse data sources, including chemical libraries, natural product datasets, biological databases and viral genomic information, are processed using AI and ML algorithms to support target identification, drug repurposing, resistance prediction, ADMET profiling and lead prioritisation. Promising candidates are subsequently subjected to computational and experimental validation to accelerate the development of novel antiviral agents.

Recent physics‐driven computational techniques including docking and MD simulations capture ligand and target contacts with atomic details; however, translating these atomic snapshots into reliable predictions of compound behaviour at cellular and organism level remains an enduring hurdle (Namba‐Nzanguim et al. 2022). This is necessary to improve the translation of computational findings to in vivo studies, where effectiveness and absorption, distribution, metabolism, excretion and toxicity (ADMET) liabilities may arise (Cherkasov et al. 2014). Several factors still limit predictive accuracy as most modelling workflows rely on relatively small, sometimes noisy datasets that span only a narrow slice of chemical space and may carry forward experimental measurement errors (Zhao 2017; Fourches et al. 2010; Stouch et al. 2003), while only a small fraction of the resulting predictions is ever validated prospectively in the wet lab (Tropsha 2010). Also, an assumption suggesting that molecules with comparable chemical structures and target activity would have similar activities could be a drawback (Zhang et al. 2017), as it may not accurately predict the activities of compounds when activity cliffs are present (Stumpfe et al. 2019). To overcome these hurdles, researchers are embracing data‐centric discovery pipelines enabled by AI and ML tools to represent compound effects that elude purely physics‐based simulations and to develop advanced, biologically meaningful similarity metrics between compounds (Fernández‐Torras et al. 2022; Jayatunga et al. 2022; Schneider et al. 2020). Figure 2 provides an overview of the role of AI and ML in antiviral drug discovery, illustrating how diverse data sources, including chemical libraries, natural product datasets, biological databases and viral genomic information, can be integrated through machine learning algorithms to facilitate target identification, drug repurposing, resistance prediction, ADMET assessment and lead prioritisation prior to experimental validation.

Algorithms are an integral part of ML performance in drug development; hence, selecting an algorithm tailored for the specific task is essential. Artificial neural networks (ANN) (Deng et al. 2022; Jiménez‐Luna et al. 2020), decision trees (DT) (Schöning and Hammann 2018; Blower and Cross 2006), support vector machines (SVM) (Rodríguez‐Pérez and Bajorath 2022) and cluster analysis (CA) (Jaeger and Banks 2023; Lund and Ma 2021; Ma et al. 2019), include the notable algorithms being used in machine learning. For instance, recently, Rajput and Kumar (2022) used SVM, random forest and AAN assessing each with tenfold cross‐validation. The study indicated that the most accurate prediction model achieved a Pearson's correlation coefficient between 0.83 and 0.98 on the T274 train/test set. Afterwards, robustness was examined using a Williams plot and the final models were subsequently incorporated into a web server.

Five algorithms, random forest (RF), SVM, naïve Bayes (NB), k‐nearest neighbour (kNN) and logistic regression, were employed to create predictive models that identify putative inhibitors of the EBOV glycoprotein and VP40 (Adams et al. 2022). In this investigation, EBOV entry inhibitors from PubChem were used to train RF, LR and SVM, yielding overall accuracies of 0.89, 0.84 and 0.86, respectively. The models were later packaged as EBOLApred, which reports a confidence score for every prediction derived from the applicability domain framework (Adams et al. 2022). Together, these results emphasise the essential significance of machine learning in the identification of drugs for Ebola virus (EBOV), indicating the need for more investigation.

The efficacy of AI and ML in antiviral discovery has been increasingly substantiated by studies that include experimental validation. Huang et al. (2021) utilised biological activity‐based machine learning models to predict compounds with anti‐SARS‐CoV‐2 efficacy, leading to the identification of about 100 candidate molecules for experimental assessment, of which 32% demonstrated verified antiviral activity in live‐virus assays, with several compounds exhibiting nanomolar potency. Gawriljuk et al. (2021) developed machine learning models utilising SARS‐CoV‐2 inhibitory datasets to prioritise drugs for in vitro testing, resulting in the identification of experimentally validated antiviral candidates. These results illustrate that AI and ML can transcend predictive modeling to facilitate experimentally informed antiviral lead development; therefore, diminishing screening expenses and accelerating the identification of promising drug candidates. However, these studies also highlight an important distinction between computational prediction performance and experimentally validated antiviral activity. Models that achieve high accuracy during training or cross‐validation may not necessarily translate into successful antiviral candidates, underscoring the importance of independent validation and prospective experimental testing.

Currently, deep learning has emerged as the predominant aspect of machine learning and is a developing method for speeding up the prediction of small molecules that are therapeutically relevant (Nag et al. 2022; Carpenter et al. 2018; Goh et al. 2017). Deep learning techniques are often used in pattern recognition problems for their strong resilience, impressive performance and straightforward structure, making them very effective in addressing nonlinear classification difficulties (Kwofie et al. 2023; Chen et al. 2018). Deep learning algorithms have been leveraged to forecast ligands of pathogens' receptors (Karki et al. 2021; Zhang et al. 2021) with the emergence of SARS‐CoV‐2, which has increased the urgency and importance of finding highly effective drugs to suppress the virus's activity (Beck et al. 2020; Bung et al. 2020; Zhang et al. 2020).

Also, a new deep learning platform named Deep Docking (DD) was developed for structure‐based virtual screening to sift through 1.3 billion molecules to discover 1000 prospective candidates against the SAR‐CoV‐2 main protease (Mpro) (Ton et al. 2020). While experimental confirmation is necessary for these anticipated molecules, they form a priority list for subsequent development. Another study used HIV‐1 genomic sequence data with resistance profiles for 18 antiretroviral treatment (ART) medicines to train deep learning models for resistance prediction in which a convolutional neural network outperformed both the multilayer perceptron and bidirectional recurrent neural networks (Steiner et al. 2020).

Deep learning models are successful in predicting viral inhibitors; however, they have certain limitations, such as the absence of standardised, open‐source workflows for repeatability. Owing to their intricate and difficult architectures, reproducing the results of a previously conducted study is often difficult (Isdahl and Gundersen 2019) and validating the conclusions of a study or replicating a comparable study in a different setting could be a notable obstacle for forecasting inhibitors because precise and consistent outcomes are essential. In addition, deep learning models are computationally demanding, necessitating substantial computer resources and time‐consuming training (Thompson et al. 2020). Choosing the appropriate machine learning (ML) model from a range of predictive models may be a challenging endeavour as the effectiveness of a model might fluctuate based on the objective and data set. Several models may exhibit strong performance on several criteria, including accuracy, precision, recall, *F*1 score and AUC‐ROC (Dinga et al. 2019). Certain models may exhibit strong performance on a certain subset of the data but demonstrate inadequate performance on the other portions, highlighting the need to account for the underlying distribution and feature characteristics. It is also important to account for trade‐offs among metrics; for example, a model with high precision might yet exhibit low recall. Therefore, when choosing a model, it is vital to carefully analyse the requirements and objectives of the work at hand (Dinga et al. 2019) and have comprehensive details of the data and job at hand and the specific metrics pertinent to the task. Consequently, priority must be given to building high‐quality and openly accessible antiviral datasets, integrating physics‐based and data‐driven models and validating computational predictions through wet‐laboratory studies. In addition, there is a need to develop reproducible and transparent workflows, leverage transfer learning across virus families and integrate AI predictions with ADMET profiling to accelerate the discovery of novel antivirals.

### 3.5 The Emergence of Gene‐Editing Technologies

Developing drugs from therapeutic compounds is difficult because viruses often exploit the host cellular machinery to replicate and spread; hence, therapeutic agents that hinder virus replication are likely to disrupt the host's normal biological processes (Rotondo et al. 2021). Moreover, the considerable variations in molecular features across viruses have posed substantial challenges in the development of comprehensive virus‐targeted treatment medicines (Mehta et al. 2021; Adalja and Inglesby 2019). Due to these reasons, the current development of antiviral drugs has switched its attention towards alternative tactics, namely the use of RNA interference (RNAi)‐based strategy (Aghamiri et al. 2019; Levanova and Poranen 2018). Aside from structural proteins, nucleic acid is critical for the assembly of an infectious virion. Consequently, the completion of the viral life cycle within host cells relies on the effective duplication of the viral genetic material. Therefore, targeted editing of viral genetic components could potentially be the most effective antiviral strategy.

Interestingly, genome editing technologies enable the modification of defined sections within the genetic material. These tools provide novel strategies for treating viral infectious illnesses by manipulating DNA sequences in the genome via addition, removal or alteration (Zhang and Li 2021). The primary genome editing techniques include zinc finger nucleases (ZFNs), transcriptional activator‐like effector nucleases (TALENs) and clustered regularly interspersed short palindromic repeats (CRISPR) and CRISPR‐associated proteins (CRISPR/Cas) (Li et al. 2014). Owing to its user‐friendliness, target precision and versatility, the CRISPR/Cas system has become a major focus (Lee 2019; Kaminski, Bella, et al. 2016; Kaminski, Chen, et al. 2016; Liao et al. 2015). This system has six different versions (Mohanraju et al. 2016), with the most significant being the type II CRISPR/Cas9 system isolated from *Streptococcus pyogenes* . Specifically, the CRISPR/Cas9 system has been proposed as a viable method for disabling target genes and inserting genetic material at precise locations in several animal models, including nonhuman primates, mice, *Drosophila* and zebrafish (Sternberg and Doudna 2015; Hsu et al. 2014).

Nucleases were first identified as a defensive mechanism in archaeal and bacterial species and have subsequently been repurposed as precise genome‐editing instruments for manipulating nucleic acid sequences in different organisms (Mojica and Rodriguez‐Valera 2016). They have the capability to alter both viral and host genomes by modifying the sequences in SDNA as well as RNA (Escalona‐Noguero et al. 2021) and can alter many genes concurrently with high‐efficiency rates (Zhou et al. 2014). However, relative to other sequence‐specific endonucleases like TALENs and zinc‐finger nucleases (ZFNs), the CRISPR‐Cas system does not require the engineering, selection and validation of target site‐specific protein pairs (Janusz et al. 2020). Instead, it utilises tiny RNAs that are simpler to design and more cost‐effective to manufacture for its targeted cutting ability, together with its high specificity and adaptability, making it an optimal therapeutic choice for viral infections (Najafi et al. 2022).

Viral infections may be lytic, latent or chronic and in the lytic phase the virus replicates actively and produces progeny virions. After the viral genetic material is enclosed in the protein shells, the particles exit the host cell via lytic rupture, which culminates in host‐cell death. Some viruses instead establish latency, entering a dormant state in which they remain transcriptionally silent for prolonged periods (Nehme et al. 2019; Traylen et al. 2011). Several human viruses, including HSV‐1, Epstein–Barr virus (EBV), cytomegalovirus (CMV), human herpesvirus 6 (HHV‐6) and HBV, can cause latent infections (De Leo et al. 2020). Chronic infection is distinguished by continuous, long‐term viral replication at a low level, leading to gradual organ damage. Features of chronic infection are exhibited in significant human viruses such as HBV and HIV‐1 (Tsukuda and Watashi 2020; Fenwick et al. 2019).

The elimination of viruses during latent and chronic states is challenging because the viral genetic material remains within host cells, as independent minichromosomes or integrated into the host DNA. Interestingly, CRISPR‐based tools can accurately target the intracellular viral genome, preventing the virus from transcribing and replicating. Thus, the CRISPR‐Cas9 system has seen broad application against DNA [HSV‐1, EBV, human papillomavirus (HPV)] and RNA [HIV, SARS‐CoV‐2] viruses. Scott et al. (2017) effectively deactivated the covalently closed circular DNA (cccDNA) in HBV‐infected hNTCP‐HepG2 cells by using the CRISPR/Cas system to specifically target the S open reading frame (ORF) of HBV. Using CRISPR/Cas9, a 2175 bp HBV insert integrated within infected cells was removed (Li et al. 2017). Adeno‐associated viral (AAV) vectors, such as AAV8 and its variants, have shown a strong affinity for the liver in these trials, thereby making them a very favourable choice for delivering the CRISPR/Cas anti‐HBV treatment.

Furthermore, CRISPR‐Cas complexes have the capability to focus on RNA viruses and at least two RNA‐directed CRISPR‐Cas approaches are now available, one of which utilises the Cas9 protein, though it does not naturally target RNA. Nevertheless, if a specific DNA oligomer containing the protospacer adjacent motif (PAM) may bind to the target RNA, forming a double‐stranded target that is necessary for Cas9‐mediated cleavage (O'Connell et al. 2014). The second platform employs Cas13, an RNase‐active effector that cleaves RNA directly (Kim 2018; Abudayyeh et al. 2017) and is under investigation as a therapeutic countermeasure against RNA viruses (Nguyen et al. 2020). Recent advances have expanded CRISPR‐based antiviral strategies beyond DNA editing to include programmable RNA targeting. Notably, the Prophylactic Antiviral CRISPR in huMAN cells (PAC‐MAN) platform demonstrated that Cas13 could target and degrade conserved regions of SARS‐CoV‐2 and influenza viral genomes, highlighting its potential as a broad‐spectrum antiviral approach (Abbott et al. 2020). Furthermore, Blanchard et al. (2021) showed that mRNA‐encoded Cas13a reduced SARS‐CoV‐2 and influenza virus infection in rodent models, providing important proof‐of‐concept evidence for the therapeutic application of RNA‐targeting CRISPR systems in vivo. These studies underscore the growing versatility of CRISPR technologies for antiviral intervention while highlighting the need for efficient delivery systems and rigorous safety evaluation prior to clinical translation. Table 3 highlights present developments in the use of genome editing technologies for antiviral therapy.

**TABLE 3:** Summary of recent studies in the use of genome editing tools for antiviral therapy.

Virus	Genome editing tool	Targets	References
Epstein–Barr virus	CRISPR/Cas9	BART, LMP1, LMP2A	Huo and Hu (2019), Van Diemen et al. (2016), Yuen et al. (2015)
Hepatitis B virus	CRISPR/Cas9	cccDNA	Seeger and Sohn (2014)
	CRISPR/Cas9	HBV core and surface proteins	Lin et al. (2014)
	CRISPR/Cas9	Core, polymerase and X ORFs	Ramanan et al. (2015)
	CRISPR/Cas9	Conserved regions of the virus	Dong et al. (2015)
	CRISPR/Cas9	Surface antigen (HBsAg)‐encoding region of the virus	Zhen et al. (2015)
	CRISPR/Cas9	DNA fragment	Li et al. (2017)
	CRISPR/Cas9	S open reading frame of HBV	Scott et al. (2017)
Human immunodeficiency virus (HIV)	ZFN	CCR5 gene	Tebas et al. (2014)
	CRISPR/Cas9	LTR, Gag	Kaminski, Bella, et al. (2016)
	CRISPR/Cas9	Proviral DNA fragment	Bella et al. (2018)
	CRISPR/Cas9	Proviral SIV DNA fragment	Mancuso et al. (2020)
	CRISPR/Cas12a	Relatively conserved HIV sequences including LTRs	Gao et al. (2020)
	CRISPR/Cas13a	Conserved regions	Yin, Zhao, et al. (2020)
Human papillomavirus HPV	CRISPR/Cas9	E6 and E7 oncogenes	Kennedy et al. (2014)
	CRISPR/Cas9	HPV16 E7 gene	Hu et al. (2014)
	CRISPR/Cas9	HPV16 promoter and E6 and E7 transcripts	Zhen et al. (2014)
	CRISPR/Cas9	E7 gene	Liu et al. (2016)
	CRISPR/Cas9	E6 and E7	Jubair et al. (2019)
SARS‐CoV‐2	CRISPR/Cas13d	Conserved viral regions	Abbott et al. (2020)
	CRISPR/Cas13d	Peptide‐coding regions of the ORF1ab and S genes	Nguyen et al. (2020)

Obviously, there have been notable advancements in genome editing for antiviral therapy; however, there are still considerable obstacles that need to be overcome before its practical usage. These drawbacks encompass precise DNA site targeting, off‐target outcomes, selective sgRNA design, Cas9 nuclease efficiency, the tendency towards homology‐directed repair instead of the predominant non‐homologous end joining, viral escape induced by mutation, reduced susceptibility to the Cas9/sgRNA machinery and the choice of an appropriate delivery modality (Lino et al. 2018; Xiao‐Jie et al. 2015). The main obstacle to clinical translation of the CRISPR/Cas system is the unintended consequences, known as off‐target effects, that may occur; hence, considering this factor while conducting in vivo examinations to establish the safety profile of viral vectors is imperative (Lin et al. 2021; Kim et al. 2015). In addition, immune responses against CRISPR‐associated proteins and viral delivery vectors may present further safety concerns that require careful assessment before clinical translation. Additional significant challenge for in vivo applications is achieving high editing fidelity while simultaneously ensuring efficient, targeted deployment of the CRISPR/Cas9 machinery.

### 3.6 Drug Combination Therapy

Combination therapy entails the combination of two or more therapeutic agents to manage a clinical condition. However, drug combinations can result in contrasting pharmacodynamic outcomes ranging from antagonistic interference or amplified toxicity to synergistic or additive therapeutic benefits (Bobrowski et al. 2021). While drug combinations could result in adverse effects owing to drug–drug interactions, their careful usage can yield positive outcomes. Combination therapy can enable concurrent modulation of multiple pathways to promote pharmacologic synergy, resulting in therapeutic gains beyond simple additive effects. Besides therapeutic effectiveness, synergy permits dose‐sparing of each component, improving tolerability and lowering toxicity. Combination therapy is applied to complex, multifactorial diseases that are hard to control, including cancer, hypertension and intractable bacterial or fungal infections (Liu et al. 2025; Campbell et al. 2022; Makhoba et al. 2020; Worthington and Melander 2013).

The therapeutic benefits of antiviral combination therapy are mostly due to pharmacological synergy and the inhibition of resistance development (Chou 2006; Cihlar and Fordyce 2016). Synergy arises when medicines targeting different viral or host elements yield a cumulative antiviral impact that exceeds the total of their separate activity (Chou 2006). Such interactions may occur through the concurrent inhibition of many stages of the viral life cycle, encompassing viral entrance, genome replication, protein processing and virion assembly (Perelson et al. 2012). Combination therapy not only enhances antiviral efficacy but also diminishes the probability of resistance development, as viruses must have numerous simultaneous changes to circumvent medicines with distinct modes of action (Peters and Conway 2011; Cihlar and Fordyce 2016). This concept is crucial in managing rapidly evolving RNA viruses, as large mutation rates enable the swift selection of drug‐resistant genotypes during monotherapy (Peters and Conway 2011). Thus, integrating antivirals with synergistic mechanisms can enhance therapeutic longevity while maintaining treatment efficacy over extended durations (Cihlar and Fordyce 2016).

HIV infection remains a chronic disease with no curative therapies or licensed vaccines; nevertheless, major advances in treatment have enabled sustained reductions in plasma viral load as well as AIDS‐related deaths. In 1987, azidothymidine, a nucleoside reverse transcriptase inhibitor (NRTI), became the first antiretroviral to receive approval for HIV treatment with consequent reduced death and infection rates among patients with AIDS (Fischl et al. 1987). From 1987 to 1995, mono or dual NRTI therapy was the common mode of treatment; nevertheless, this treatment mode was either suboptimal or ineffective (Akanbi et al. 2012; Peters and Conway 2011). Monotherapy failure was largely driven by resistance, reflecting HIV's high mutation rate typical of RNA viruses due to the error‐prone absence of proofreading during reverse transcription (Peters and Conway 2011). Consequently, protease inhibitors (PI) were introduced into the HIV treatment regimen, prompting a substantial shift in the standard of care. Therefore, the recognised standard treatment for HIV infections is the combination antiretroviral therapy (cART) consisting of two NRTIs and a PI, which has resulted in substantial reductions in HIV infection, progression to AIDS, secondary infections and death rates (Cihlar and Fordyce 2016; Akanbi et al. 2012; Peters and Conway 2011). HIV infection is now a controllable chronic condition with life expectancy comparable to the general population because of cART (Samji et al. 2013).

Just like HIV, no effective vaccines exist for HCV; nevertheless, antiviral medications achieve cures in about 95% of cases, thereby lowering mortality risk due to HCV‐related comorbidities (WHO 2024). In the last two decades, HCV has been managed with non‐PEGylated and subsequently PEGylated interferon (IFN), either in combination with or without ribavirin (Parlati et al. 2021). Unfortunately, poor clinical results were the outcomes of IFN/RBV treatment with a < 50% cure rate contingent upon HCV genotypes and other comorbidities (Parlati et al. 2021; Kish et al. 2017). In 2011, the standard shifted to a triple regimen that added first‐generation NS3/4A protease inhibitors such as telaprevir or boceprevir, lifting SVR rates to about 75% (Pawlotsky 2013). Furthermore, cure rates rose to roughly 95% without an uptick in noticeable side effects after direct acting antivirals (DAAs) were introduced, notably sofosbuvir, a uridine nucleotide prodrug that targets the HCV NS5B polymerase and daclatasvir, an NS5A inhibitor (Parlati et al. 2021). Consequently, combination therapy shortens treatment courses while sustaining efficacy and limiting adverse events. It also offers a practical means to combat drug resistant HCV in patients presenting with additional comorbidities. Recent reports show improved response rates when novel drug cocktails were given to DAA experienced individuals who had relapsed (Belperio et al. 2019; Sarrazin et al. 2018; Bourlière et al. 2017). Remarkably, co‐administering antiretrovirals devised for HIV/HCV coinfection produced robust HIV viral suppression and elevated HCV SVR without discernible toxicity (Huhn et al. 2020).

The rapid mutation rate of influenza viruses makes drug resistance possible and poses therapeutic difficulties. During the 1980s and early 2000s, extensive resistance to the adamantanes amantadine and rimantadine emerged, resulting in worldwide resistance in 45% of influenza A infections (Nguyen et al. 2010). While most influenza strains remain vulnerable to several neuraminidase inhibitors (Melville et al. 2018; Hussain et al. 2017), nearly universal oseltamivir resistance (98%–100%) was reported among circulating viruses in the 2008–2009 season (Melville et al. 2018). To address this problem, multidrug regimens may be explored as the simultaneous evolution of resistance to multiple antivirals is comparatively unlikely (Perelson et al. 2012). Recently, Wang et al. (2020) showed that combination treatment may expedite recovery relative to oseltamivir only after evaluating the efficacy of the combination of a favipiravir‐oseltamivir regimen against severe influenza. Generally, substantial advancement has been reported in the combination of free drugs for disease treatment (Komarova and Boland 2013); nevertheless, differences in pharmacokinetics and membrane transport between agents, together with dosing and scheduling complexity, create challenges for combination therapy and can yield results that fall short of therapeutic impacts (Hu et al. 2010). Unfortunately, significant adverse side effects could result from the concurrent use of highly potent drugs (Aditya et al. 2013). Thus, subsequent investigations should concentrate on designing antivirals mediated by resistance modelling, developing fixed‐dose or co‐delivery systems to optimise drug exposure and assessment of triple or mixed regimens that incorporate direct‐acting and host‐directed agents to boost efficacy reduce toxicity and limit resistance.

### 3.7 Nanotechnology‐Enhanced Drug Delivery System for Antiviral Development

The primary causes of drug resistance are mutation‐driven viral variation under drug pressure, together with poor cellular selectivity and subtherapeutic drug exposure at the site of action, hence the renewed interest in nanotechnology, which has resulted in nanomedicine delivery platforms designed for controlled, site‐directed administration to maintain therapeutic levels at diseased tissues, lower dosing frequency, improve bioavailability, limit premature metabolism, bypass efflux transporters and increase cell‐specific uptake (Pradhan et al. 2021). The unique features that make a promising drug‐delivery system include nanoscale dimensions that exploit the boosted permeability and retention effect, high surface‐area‐to‐volume ratios, straightforward surface functionalisation for ligand‐based targeting and conjugation with biocompatible moieties to prolong circulation time (Pradhan et al. 2021). Nanotechnology‐based antiviral delivery systems comprise various carrier platforms, such as lipid‐based nanoparticles, polymeric nanoparticles, metallic nanoparticles, dendrimers, nanogels and virus‐like nanoparticles, each exhibiting unique physicochemical properties that affect drug loading, release kinetics, biodistribution and antiviral efficacy (Lembo et al. 2018; Maus et al. 2021). Targeting can be accomplished via passive mechanisms that utilise the physicochemical characteristics of nanocarriers and tissue microenvironments, or through active strategies that involve surface modification with ligands, antibodies, peptides or receptor‐binding molecules to improve selective uptake by infected cells (Lembo et al. 2018; Mitchell et al. 2021). The selection of carrier and targeting approach is determined by the viral pathogen, target tissue, mode of administration, therapeutic payload and the biological obstacles that must be surmounted to attain effective antiviral activity (Mitchell et al. 2021; Maus et al. 2021).

Basically, antiviral medications are loaded within or adsorbed onto the nanoparticle matrices, protecting the drugs from immune clearance and have extended systemic residence period, hence improving their bioavailability and therapeutic effectiveness (Zeng et al. 2022; Ma et al. 2020; Kulkarni et al. 2019). Once nanoparticles (NPS) reach their target tissues, they interact with cell membranes and promote uptake through receptor engagement or targeting ligands (Donahue et al. 2019; Cullis et al. 2017; Lara et al. 2017; Xin et al. 2017; Blanco et al. 2015; Verma et al. 2008). After internalisation, drug release occurs via matrix degradation, receptor‐triggered unloading or stimulus‐responsive mechanisms dictated by the local microenvironment (Chen and Feng 2022; Islam et al. 2021; Vaughan et al. 2020; Dadfar et al. 2019; Ghorbani and Hamishehkar 2017; Uva et al. 2015).

The success of targeted antiviral delivery depends not only on tissue accumulation but also on efficient intracellular trafficking and subcellular delivery (Mitchell et al. 2021; Maus et al. 2021). Following cellular uptake, nanocarriers are commonly transported through endosomal pathways, where therapeutic payloads may be degraded unless effective endosomal escape mechanisms are incorporated (Mitchell et al. 2021). To address this challenge, several delivery systems have been engineered with pH‐responsive, membrane‐disruptive or fusogenic components that facilitate cytosolic release of antiviral agents (Maus et al. 2021; Schoenmaker et al. 2021). In addition, organ‐specific targeting has emerged as an important strategy for improving therapeutic efficacy while minimising off‐target toxicity. For respiratory viral infections, inhalable nanomedicines offer direct delivery to the lungs, enhancing local drug concentrations while reducing systemic exposure (Patton and Byron 2007; Loo et al. 2023). Similarly, surface‐functionalised nanoparticles can be designed to preferentially accumulate in organs such as the liver, a major target for chronic viral infections including hepatitis B (Lembo et al. 2018; Mitchell et al. 2021). These advances highlight the growing importance of precision delivery strategies in maximising antiviral efficacy and improving clinical outcomes.

Furthermore, the NPs delivery approach could facilitate the simultaneous delivery of multiple antiviral compounds, enabling combination delivery to boost potency and curb resistance (Li et al. 2022). They can disrupt the interaction at the virus‐receptor interface by incorporating targeted ligands or antibodies via surface functionalisation by obstructing entry into host cells. The surface binding sites of NPs can engage target biomolecules to inactivate viruses. In addition, metals such as gold, silver, copper and zinc are internalised by innate immune cells, notably macrophages and dendritic cells, thereby triggering activation in support of host defence. Metal nanoparticles have shown broad‐spectrum virucidal activity against multiple viruses including HIV‐1, influenza, hepatitis B and HSV (Galdiero et al. 2011).

The molecular basis for the antiviral effect of transition metals entails several pathways ranging from blocking viral entry to chemically inactivating intact virions (Singh et al. 2017). Specifically, metal NPs bind viral surface proteins via Kazimir forces, van der Waals interactions and disulphide exchange (Demchenko and Rusinchuk 2019; Yadavalli and Shukla 2017). For instance, silver nanoparticles (AgNPs) and gold nanoparticles (AuNPs) can break viral surface proteins disulphide bonds on the sulfhydryl groups, thereby blocking viral entry (Jeremiah et al. 2020; Kim et al. 2020). Cagno et al. (2018) developed NPs equipped with long flexible linkers that mimic heparan‐sulfate proteoglycans; the multivalent attachment of these repeating motifs exerts forces of 190 pN, irreversibly deforming virions (Reina et al. 2020). Likewise, porous gold nanoparticles covalently tethered to hyaluronic acid via Au–Sulphide bonds hinder membrane fusion and subsequent internalisation, rendering influenza virus non‐infectious (Kim et al. 2020).

In addition, NPs can exert intracellular antiviral activity by directly associating with viral DNA or RNA. For instance, AgNPs inhibit viral reverse transcription by complexing with sulphur and oxygen bearing thiol, phosphate and amino groups on nucleic acids or by attaching directly to the genome itself (Galdiero et al. 2011). Also, glutathione (GSH)‐capped Ag_2_S has been reported to block the production of negative‐sense viral RNA and the antiparasitic agent ivermectin has also been reported to hinder replication of viral genomes. A clinical trial reported safety and efficacy of an intranasal ivermectin mucoadhesive nanosuspension in patients with mild COVID‐19, with rapid improvement in respiratory symptoms, including anosmia, cough and dyspnoea, improved rapidly following treatment (Aref et al. 2021). However, these clinical outcomes alone do not confirm that symptom improvement directly resulted from the inhibition of viral genome replication.

Furthermore, NPs facilitate the induction of IFN‐stimulated genes (ISGs) and pro‐inflammatory cytokines, blocking porcine epidemic diarrhoea virus infection (Du et al. 2018). Nanovectors can obstruct the virus‐receptors encounter, thus preventing viral entry using mechanisms like receptor mimicry, steric hindrance and chemical competition. Receptor mimicry involves the design of a nanocarrier that possesses structural or surface properties like those of the viral ligand, facilitating the nanocarrier's binding to the virus and stimulating its interaction with the receptor. The nanocarrier can inhibit normal interaction by binding to the virus competitively. For example, AgNPs inhibit the HIV gp 120‐CD4 association and curb infection by interacting with sulphur and oxygen‐bearing thiol and phosphate groups on nucleic acids and proteins or binding to DNA or RNA directly to slow the viral reverse transcription rate (Galdiero et al. 2011). Similar to nucleic acid inhibitors, long‐acting rilpivirine (RPV LA) nanosuspension produced prolonged (> 4 months) suppression of HIV infection in human rectal tissue explants, as evidenced by a Phase 1 study (*p* < 0.0001) (NCT01656018) (McGowan et al. 2016). Positively charged ZnO NPs, in turn, block SARS‐CoV‐2 and host cell receptors, thereby disrupting virus‐host attachment (Alavi et al. 2022; Hamdi et al. 2021).

The nano carrier's presence can result in the formation of additional space, competing with virions for receptor‐binding sites, consequently, the virus cannot bind effectively with the host's cell receptor. NPs can trigger targeted immune responses to enhance viral identification and elimination by immune cells and can activate pattern recognition receptors (PRRs) and leading to interferon (IFN) induction. NP surface architecture and composition can directly engage PRRs, thereby initiating downstream signalling. Montague et al. (2021), observed that surface‐charged NPs interact through electrostatic forces, promoting association with and activation of platelet glycoprotein receptors, effectively acting as PRR mimetics for host and pathogen derived charged ligands. Conversely, some nanocarriers are also internalised by immune cells; once inside, their released components engage intracellular PRRs and trigger signalling cascades that activate the cells and amplify immune responses. Remarkably, certain nanomaterials stimulate PRRs without phagocytic uptake. For example, Yazdi et al. (2010), showed that titanium‐dioxide NPs directly activate the NLRP3 inflammasome, driving intracellular IL‐1α and IL‐1β release and contributing to pulmonary inflammation.

Furthermore, NPs may regulate immune responses by inhibiting and overproducing reactive oxygen species (ROS). Viral infection‐mediated intracellular ROS inhibits natural immunity; thus, inhibitors that reduce ROS levels may effectively halt viral proliferation. Glycyrrhizic‐acid‐based carbon dots regulate intracellular ROS levels to hinder porcine reproductive and respiratory syndrome virus (PRRSV) replication (Tong et al. 2020). Also, oseltamivir‐modified AgNPs significantly lowered phosphorylated p53 and total p53 levels in MDCK cells via engagement of ROS‐dependent AKT and p53 signalling pathways when their capacity to disrupt viral activity was investigated (Li et al. 2016). Therefore, the utilisation of nanotechnology in antiviral research extends beyond enhancing the delivery of antiviral agents; it also involves the blocking of viral infection and immune activation, providing fresh therapeutic avenues and renewed prospects for the treatment of viral diseases.

Despite numerous advantages linked with the application of nanotechnology for antiviral therapy, some challenges need to be addressed for optimal application of this technology. Toxicity is one of the topical issues that must not be disregarded as metal NPs exhibit toxicity, resulting in mitochondrial and DNA damage (Xiong et al. 2022; Bhargava et al. 2021; Buchman et al. 2019). Organs characterised by rapid perfusion, including the renal, hepatic, pulmonary and splenic organs, are vulnerable to NP accumulation and resultant damage (Mitchell et al. 2021; Li et al. 2020). Particle size is a crucial determinant affecting nanotoxicity (Liu et al. 2022; Egbuna et al. 2021); hence, manipulating the size and morphology of NPs can reduce their toxicity and subsequently influence their biodistribution, degradability and cellular internalisation properties.

Another challenge is the existence of various barriers in humans, such as blood circulation, immune system clearance and cell membrane barriers, all of which may restrict NPs delivery efficiency and site‐specific targeting. For instance, the most utilised method of drug administration is oral delivery; unfortunately, the acidic environment and several degradative enzymes residing in the gastrointestinal tract (GI) might impair absorption, resulting in low stability and poor bioavailability (Ejazi et al. 2023; Wang et al. 2023; Beatty and Lewis 2019; Poon et al. 2019; Desai 2012). Consequently, it becomes cumbersome for such drugs to reach therapeutic levels in the bloodstream; thus, there is a need for dedicated research on non‐intrusive or less intrusive techniques for their administration.

The stability of nanomaterials during preparation, storage and application is influenced by the production method, environmental exposures (thermal conditions, humidity and light) and in vivo factors (pH and enzymatic activity) and is a significant concern (Ruan et al. 2022; Verma et al. 2021; Chen et al. 2019). Instability could result in increased toxicity and other negative effects as they accumulate or deteriorate in the environment, thereby impacting their efficacy and long‐term performance. Specifically, ivermectin exhibits significant antiviral action against the zika virus; however, further progress on its nano drug is constrained by poor water solubility and the potential impact of specific formulations on its pharmacokinetic profile, which may alter plasma kinetics (Caparco et al. 2023; Surnar et al. 2019). Additionally, some NPs not toxic in the short term could become toxic with prolonged exposure; hence, modifying the production procedure or developing a stabiliser could address these issues (Tian et al. 2022; Kang et al. 2018).

Due to their distinctive features and accompanying risks, the application of nanomaterials encounters regulatory and ethical challenges; robust oversight frameworks must therefore be instituted to ensure safety and adherence to standards. In light of prospective clinical translation, formulations containing NPs should be manufactured in strict accordance with established specifications (Yu et al. 2019). Consequently, rigorous and standardised evaluation of nanodrug efficacy, safety, quality, large‐scale manufacturability, long‐term stability and regulatory compliance is essential to facilitate successful clinical translation and widespread implementation of nanotechnology‐based antiviral therapies.

## 4 Translational Integration: Linking Natural Products, Computation and Delivery

**FIGURE 3:**
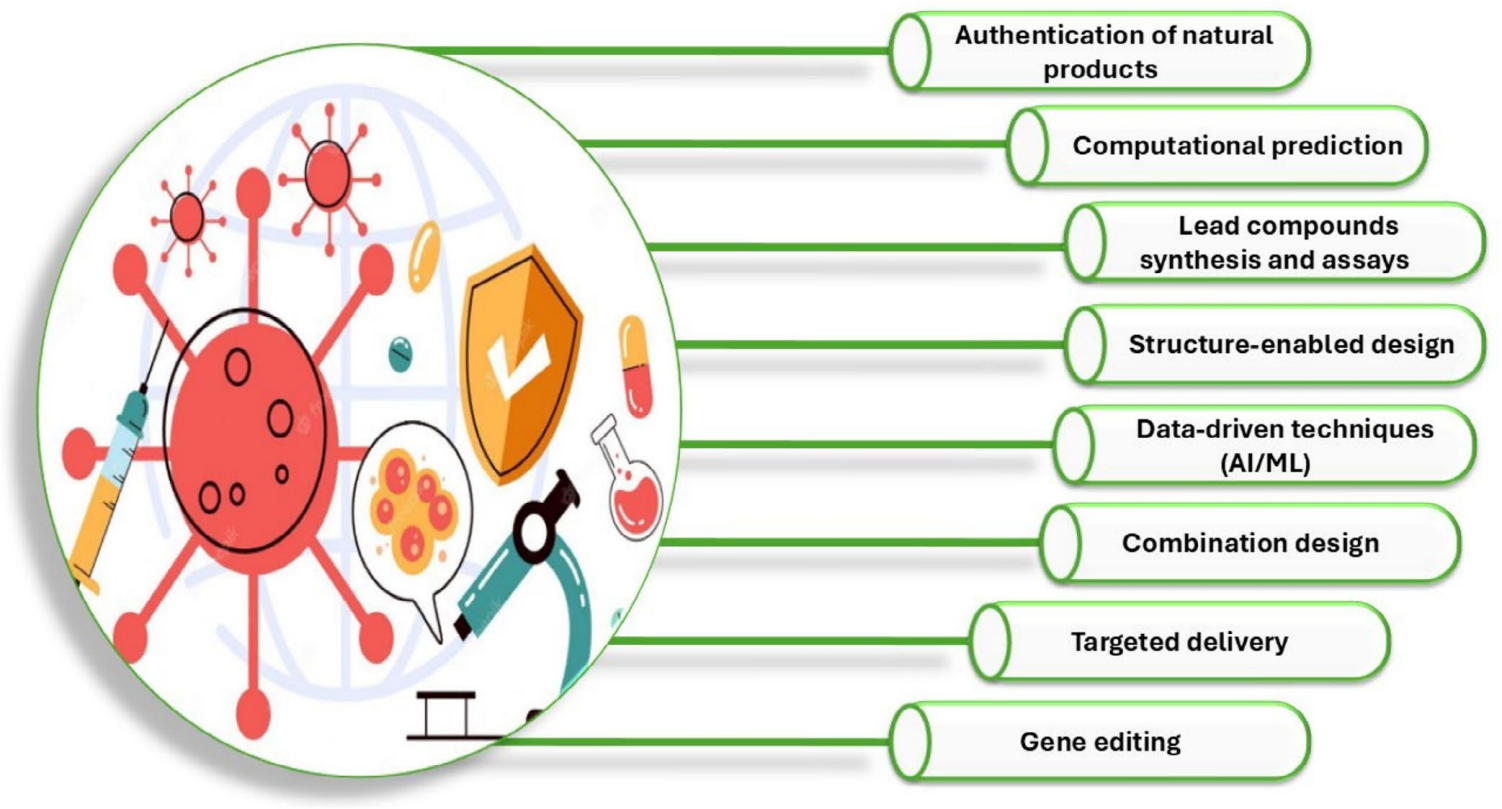
Pathway for integrating natural products, computation and delivery into antiviral designs.

Considering the highlighted drawbacks of conventional antivirals, a stepwise translational framework linking natural‐product discovery, computational prioritisation, experimental validation, lead optimisation and targeted delivery is proposed to advance antiviral design (Figure 3). Firstly, there is a need for a proper authentication of plant‐derived compounds via purity assessment and dereplication. Thereafter, computational methods support scaffold hopping and screening by proposing tractable cores and ranking candidates against property and mechanism filters, while synthesis and assays remain the means of establishing structure and activity (Adeosun and Loots 2024; Atanasov et al. 2021). After hit validation, next is structure‐enabled design which guides optimisation. At this juncture, X‐ray or cryo‐EM can be adopted when an experimentally verified binding pose will alter substituent vectors, as demonstrated in recent discovery of viral protease inhibitors (Cebi et al. 2024; Unoh et al. 2022; Owen et al. 2021). Data‐driven discovery approaches including AI and ML are useful when run prospectively with blinded hold‐out data and open reporting of errors. Otherwise, they should guide models' hypothesis generation and not control critical decisions (Serrano et al. 2024). Computation is most credible when its processes are repeatable and when models are related to structural constraint while pre‐specified metrics promote transparent assertions of synergy or additivity; however, post hoc selection of metrics undermines inference (Ianevski et al. 2020).

When efficacy of the drug candidate is limited by exposure, then delivery should be engineered to the site of action hence conventional formulations should be applied when they achieve target exposure. Therefore, enhancement with nanotechnology systems is justified in cases where in vivo biodistribution and release can be quantified and connected to pharmacodynamic effect (Okada et al. 2024; Supramaniam et al. 2023). Similarly, these criteria are applicable to nucleic‐acid therapeutics and gene‐editing strategies as advancement necessitates defined on‐ and off‐target profiles and verified tissue delivery (Christensen et al. 2025; Azeez et al. 2024) (Figure 3). Overall, natural products should be authenticated, purified and dereplicated before any modelling. Structural methods such as X‐ray or cryo‐EM are most valuable when a verified pose will change substituent orientation or warhead choice. Both CADD and data‐driven approaches should be validated on blinded data with transparent error reporting. Ultimately, combination studies should declare a primary synergy model in advance while advanced delivery systems are justified only when they address a measured exposure deficit at the site of action.

## 5 Conclusion and Future Perspectives

The recent pandemic underscores the imperative to advance broad‐spectrum antivirals to effectively address the accompanying health crises and combat emerging viral diseases. Additionally, the tendency of viruses to develop resistance to the available medications presents a notable public health issue, while their capacity for genome modification complicates the production of effective treatments and antivirals. Therefore, the development of efficient and cost‐effective antivirals continues to pose a significant challenge. This review outlines the challenges associated with conventional antiviral drugs and examines several emerging strategies for the design and advancement of next‐generation antiviral compounds to improve resistance barriers and therapeutic potency. Nonetheless, these approaches exhibit certain limitations that need to be addressed, while the combinatorial approach of these emerging technologies may be more effective in addressing these issues. Thus, incorporating medicinal chemistry with bioinformatics, artificial intelligence, gene‐editing and nanotechnology may facilitate the identification of new therapeutic indications. The implementation of high‐throughput screening together with rigorous safety and performance evaluation pipelines will accelerate antiviral development.

However, the successful translation of antiviral platforms into clinically accessible therapies will rely on both scientific innovation, scalability and manufacturability. Emerging technologies, such as gene‐editing technologies and nanotechnology‐enhanced drug delivery systems, typically necessitate specialised infrastructure, advanced manufacturing methods, rigorous quality‐control measures and significant financial commitment. These requirements may pose considerable obstacles in low and middle‐income nations, where restricted manufacturing capabilities, reliance on imported technologies, supply chain vulnerabilities and regulatory limitations all hinder access to novel antiviral therapies. Consequently, future research must prioritise scalable production methods, economical formulations, knowledge transfer activities and regulatory harmonisation to ensure equitable global access to next‐generation antiviral therapies. In light of this, a simple, decision‐focused pathway integrating natural products, computation and delivery to guide choices from discovery through optimisation and translation prior to another possible viral pandemic was outlined. Despite their considerable promise, many of the emerging antiviral strategies discussed in this review remain constrained by challenges related to reproducibility and validation, toxicity and biosafety concerns and uncertainties regarding long‐term clinical efficacy. Mitigating these constraints through rigorous validation, standardised evaluation frameworks and extensive clinical investigations will be critical to establishing their therapeutic efficacy and facilitating successful clinical translation. Arising from the data garnered in this review, future research needs to harness a combination of emerging technologies to enhance the discovery and optimisation of effective antiviral therapeutics, to address current viral infections and to strengthen preparedness to avert future pandemic emergence.

## Author Contributions

**Feroz Mahomed Swalaha:** writing – review and editing, funding acquisition, supervision. **Saheed Sabiu:** conceptualization, funding acquisition, supervision, writing – review and editing. **Adedayo Ayodeji Lanrewaju:** conceptualization, writing – original draft, writing – review and editing. **Abimbola Motunrayo Folami:** funding acquisition, writing – review and editing, supervision.

## Funding

This work was supported in part by the South African Medical Research Council, National Research Foundation of South Africa (Grant SRUG2204193723) and Water Research Commission (K5/C2020‐2021‐00181).

## Conflicts of Interest

The authors declare no conflicts of interest.

## Data Availability

The data are contained within the article.
